# Ultra-Rapid Lispro Improves Postprandial Glucose Control and Time in Range in Type 1 Diabetes Compared to Lispro: PRONTO-T1D Continuous Glucose Monitoring Substudy

**DOI:** 10.1089/dia.2020.0129

**Published:** 2020-11-09

**Authors:** Maciej T. Malecki, Dachuang Cao, Rong Liu, Thomas Hardy, Bruce Bode, Richard M. Bergenstal, Juliana Bue-Valleskey

**Affiliations:** ^1^ Department of Metabolic Diseases, Jagiellonian University Medical College, Krakow, Poland.; ^2^ DC and RL Clinical Design, Delivery, and Analytics, TH and JB-V Connected Care and Insulins—Medical Development, Eli Lilly and Company, Indianapolis, Indiana, USA.; ^3^ Endocrinologist and Partner, Atlanta Diabetes Associates, Atlanta, Georgia, USA.; ^4^ Endocrinologist and Executive Director, International Diabetes Center, Minneapolis, Minnesota, USA.

**Keywords:** Ultra-rapid lispro, Type 1 diabetes, Continuous glucose monitoring, Time in range

## Abstract

***Background:*** This study evaluated glucose control by continuous glucose monitoring (CGM) during treatment with ultra-rapid lispro (URLi) or lispro used in combination with insulin glargine or degludec in adults with type 1 diabetes in a substudy of the PRONTO-T1D study.

***Methods:*** Ambulatory glucose profiles were evaluated in 269 patients from PRONTO-T1D assigned to double-blind URLi (*n* = 97) or lispro (*n* = 99) given 0–2 min before the start of the meal (mealtime), or open-label URLi (*n* = 73) given 20 min after the meal (postmeal URLi). Blinded CGM was used for up to 14 days before baseline and the 26-week primary endpoint. The primary objective was to compare mealtime URLi and lispro with respect to incremental area under the serum glucose concentration versus time curve from 0 to 2 h (iAUC_0–2h_) after breakfast.

***Results:*** Mealtime URLi was superior in reducing the iAUC_0–2h_ when compared to lispro for breakfast (least squares mean [LSM] difference −28.1 mg·h/L, *P* = 0.048) and for all meals combined. iAUC_0–3h_ and iAUC_0–4h_ were also reduced. Postmeal URLi resulted in similar postprandial glucose (PPG) control to mealtime lispro, but less optimal PPG control compared to mealtime URLi. Mealtime URLi increased daytime time in range (71–180 mg/dL [3.9–10.0 mmo/L]) (LSM difference = +43.6 min, *P* = 0.020) and decreased nighttime time in hypoglycemia (LSM difference ≤70 mg/dL [3.9 mmol/L] = −11.5 min, *P* = 0.009) compared to mealtime lispro.

***Conclusions:*** Results of this CGM substudy support the improved PPG control seen with mealtime URLi in the PRONTO-T1D study and show that mealtime URLi resulted in improved daytime time in target range.

## Introduction

Use of rapid-acting insulin analogs results in reduced postprandial hyperglycemia when compared to human insulin.^1^ Nevertheless, elevated postprandial glucose (PPG) is a persistent challenge to diabetes management among patients with both type 1 diabetes and type 2 diabetes.^2,3^ Ultra-rapid lispro (URLi) is a novel ultra-rapid insulin lispro formulation developed to more closely match physiological insulin secretion in response to meals and improve PPG control.^4^ The URLi formulation includes two key locally acting excipients, treprostinil and citrate, which accelerate the absorption of insulin lispro from the site of injection via independent mechanisms of action. Microdoses of treprostinil in URLi induce local vasodilation, while citrate increases vascular permeability.^5,6^ URLi was efficacious with a similar safety profile to lispro (Humalog^®^) in phase 3 studies^7^ and type 2 diabetes.^8^

The PRONTO-T1D study, which evaluated the efficacy and safety of URLi versus lispro in adults with type 1 diabetes, met the primary endpoint of noninferior hemoglobin A1c (HbA1c) change from baseline compared to lispro at 26 weeks, when insulins were dosed at mealtime.^7^ Noninferiority for postmeal URLi (administered 20 min after the start of a meal) versus mealtime lispro was also shown. Mealtime URLi was superior to mealtime lispro in controlling 1- and 2-h PPG excursions during standardized test meals. Postmeal URLi provided similar PPG control during the test meal compared to mealtime lispro but was less optimal compared to mealtime URLi. The results of this trial have been published.^7^ The PRONTO-T1D study included a continuous glucose monitoring (CGM) substudy, the results of which are reported here.

The aim of this CGM substudy of PRONTO-T1D was to compare glucose control as measured by CGM during treatment with URLi or lispro, when either was used in combination with basal insulin glargine or insulin degludec. This includes incremental area under the serum glucose concentration versus time curve (iAUC) after the start of breakfast and all meals as well as time in target glucose range.

## Methods

### Study design and treatment

A detailed description of the study design and primary results has been published.^7^ In brief, this was a phase 3, treat-to-target study comparing URLi and lispro as part of a multiple daily injection regimen in adult patients with type 1 diabetes. Patients were treated with either insulin glargine or insulin degludec throughout the study in combination with prandial insulin. Following an 8-week lead-in period for basal insulin optimization, patients were randomized to one of three groups and were permitted to use carbohydrate counting or pattern adjustment to manage prandial insulin dosing requirements. In two of the treatment groups, URLi and lispro were administered immediately (0–2 min) before each meal (mealtime) in a double-blind manner.

A third open-label treatment group consisted of URLi administered 20 min after the start of a meal (postmeal URLi). The study was designed to demonstrate noninferiority of URLi, compared with lispro in change from baseline to week 26 in HbA1c, when URLi or lispro was administered at the start of the meal. Blinded CGM was offered to a subgroup of patients at selected sites in PRONTO-T1D. It was not mandatory for patients to participate in the CGM substudy; however, all patients who participated in this substudy had to meet the inclusion criteria for PRONTO-T1D and participated in all study visits and procedures.

Adult patients with type 1 diabetes, diagnosed based on the World Health Organization criteria,^9^ and continuously using insulin for ≥1 year were eligible for participation if treated with a rapid-acting insulin analog ≥90 days and basal insulin ≥30 days before screening, with an HbA1c 7.0%–9.5% (53.00–80.32 mmol/mol) and body mass index ≤35 kg/m^2^. Patients who participated in PRONTO-T1D were randomly assigned to mealtime URLi, mealtime lispro, or postmeal URLi in a 4:4:3 randomization ratio. Stratification for PRONTO-T1D was by country, HbA1c stratum (≤7.5%, >7.5%), type of basal insulin during the lead-in period, and prandial insulin dosing plan during the study.^7^

Blinded CGM (Dexcom G4 Platinum System) was used for up to 14 days before baseline and the 26-week primary endpoint. Patients were instructed to mark the start time for each meal or snack (carb event) and the time for each insulin injection (insulin event) using the *Events* feature of the CGM system, and perform glucose calibration as recommended by the manufacturer during the CGM sessions.

The study was conducted in accordance with the International Conference on Harmonization Guidelines for Good Clinical Practice and the Declaration of Helsinki. All patients provided written informed consent.

### Statistical analyses

Raw data were collected for up to 14 days of CGM use. Prespecified criteria were used to define valid CGM days for inclusion in analysis as follows:

- For assessments of the 24-h, daytime, or nocturnal periods, at least 70% of the total measures expected within that timeframe and no missing period longer than 3 h was required.
- For mealtime assessments, at least 70% of the total measures for that meal and no missing period longer than 20 min for iAUC_0–2h_ (or 25 min for other by-meal outcome variables) was required.

The primary outcome measurement was the iAUC_0–2h_ after the start of breakfast at week 26, with iAUC_0–2h_ being the average of iAUC_0–2h_ values on all valid CGM days and iAUC_0–2h_ on each day being the total area under the glucose curve and above the glucose concentration before the start of breakfast during 0–2 h after the start of breakfast. The iAUCs for breakfast as well as midday and evening meals were calculated by applying the trapezoidal rule to both positive and negative glucose increments.

The area of each small trapezoid was first calculated and then the iAUC was derived as the total sum of those individual areas. If any trapezoid fell below the starting glucose, a negative value was obtained for its area that was in effect subtracted from the area obtained from above the starting glucose (the average of the CGM values in the time window [−19, 0] minutes relative to the start of the meal). Breakfast, lunch, and dinner were defined as the first carb event during the time intervals of 04:00–11:00 h (4 am–11 am), 11:00–16:00 h (11 am–4 pm), and 16:00–21:00 h (4 pm–9 pm), respectively, that was associated with an insulin event within ±30 min. Data from the period up to 4 h for all meals combined were also analyzed.

Assuming a 20% dropout rate for 26 weeks, ∼289 patients (105 in mealtime URLi, 105 in mealtime lispro, and 79 in postmeal URLi) were planned to participate in this substudy, providing at least 80% power to show superiority of mealtime URLi versus mealtime lispro in iAUC_0–2h_ after the start of breakfast assuming a mean difference of 27 mg·h/dL and a standard deviation (SD) of 62 mg·h/dL.

All patients who were enrolled in this substudy and were randomized to one of the study treatments, received at least 1 dose of study treatment, and had CGM data from at least 1 collection period (either baseline or endpoint) included in the analyses.

Statistical comparisons were based on a two-sided significance level of 0.05. There was no multiplicity adjustment.

For PPG-related variables (postmeal iAUC, postmeal glucose excursions), the percentage of patients with baseline missing was >20% (mostly due to missing meal event markers); thus, as prespecified, a constrained longitudinal data analysis was performed.^10,11^

Hypoglycemia/hyperglycemia rate as measured by CGM data was analyzed using a negative binomial regression model and the proportion of patients with at least 1 hypoglycemia/hyperglycemia event as measured by CGM data (incidence) was analyzed using a logistic regression model, with a hypoglycemia/hyperglycemia event defined as at least 10 consecutive minutes below/above the specified threshold and determined by 3 or more consecutive CGM values meeting the criterion. For other CGM variables (glycemic variability, average daily glucose, daily average AUC, hourly average glucose), an analysis of covariance model was used. For continuous variables, the baseline value was included as a covariate in the analysis models to account for potential baseline imbalance in this substudy, and stratification factors without severe imbalance were used as fixed effects.

The glucose thresholds prespecified for calculation of various time in range (TIR) parameters in this study were different from those recommended in newer consensus guidelines.^12^ As a result, data were reanalyzed using recent thresholds (for example TIR from 70–180 mg/dL and time in hypoglycemia <70 mg/dL and <54 mg/dL) and results of these reanalyses are presented in Supplementary Table S4 where different.

## Results

### Disposition

A total of 388 patients signed the informed consents for both the main study and the CGM substudy, and 313 of these patients were randomized. Ambulatory glucose profiles (AGPs) were evaluated in 269 patients from PRONTO-T1D assigned to mealtime URLi (*n* = 97), mealtime lispro (*n* = 99), and postmeal URLi (*n* = 73). Patient disposition was similar between treatment groups with ∼94% of patients overall completing study treatment as part of the CGM cohort (Supplementary Table S1). A detailed description of patient disposition for the main study population has been published.^7^

### Demographics and key non-CGM-related study results

Baseline characteristics were similar between treatment groups in the CGM cohort (Supplementary Table S2) and representative of the main study cohort in PRONTO-T1D.^7^

In general, the results from key glycemic control parameters in the CGM population were consistent with the full study population indicating that the CGM population was representative of the full study population (Supplementary Table S3).

## CGM Results

### Ambulatory glucose profiles

**FIG. 1.**
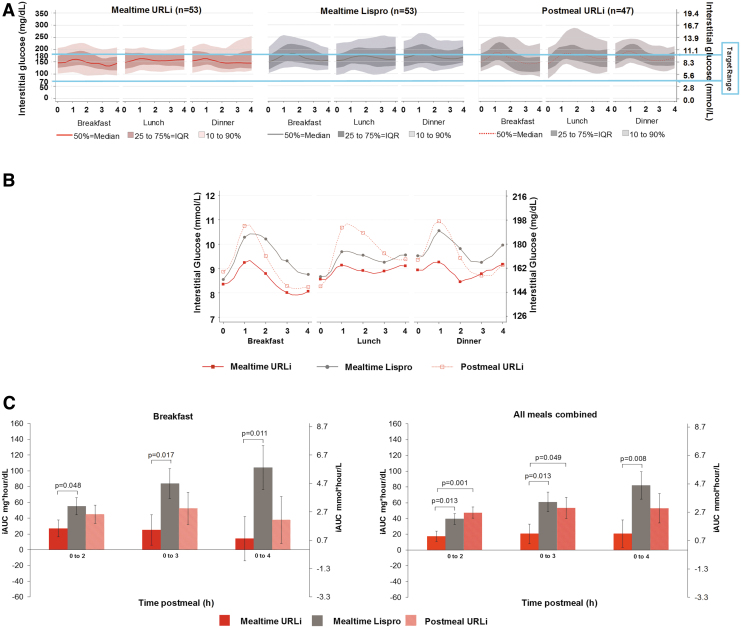
Mealtime and postmeal glucose control. **(A)** Ambulatory glucose profiles (median and percentile) by treatment group 0–4 h postmeal at week 26. **(B)** Ambulatory glucose profiles (mean) by treatment group 0–4 h postmeal at week 26. **(C)** Incremental glucose AUC postbreakfast and all postmeals combined. AUC, area under the curve; iAUC, incremental AUC; IQR, interquartile range; *n*, number of subjects who had valid continuous glucose monitoring data per prespecified criteria; URLi, ultra-rapid insulin lispro.

Figure 1A presents the AGPs by meal for each treatment group showing the median glucose control line, the 25th to 75th percentile lines, and the 10th to 90th percentile lines over 4 h after each meal. Figure 1B presents the mean glucose profile over 4 h from each meal from the AGPs. Visual examination of these AGPs showed that mealtime URLi was associated with smaller PPG excursions compared to mealtime lispro. In addition, postmeal URLi was associated with more variable PPG excursions compared to mealtime URLi and mealtime lispro.

### iAUC after meals

URLi administered immediately before breakfast resulted in a statistically significant reduction in the iAUC_0–2h_ after breakfast (least squares mean [LSM] difference −28.1 mg·h/L, *P* = 0.048) when compared to mealtime lispro and remained statistically significantly lower for both iAUC_0–3h_ and iAUC_0–4h_ (Fig. 1C). Similarly, the iAUC_0–2h_, iAUC_0–3h_, and iAUC_0–4h_ for all meals combined were statistically significantly lower in the mealtime URLi group compared to the mealtime lispro group (Fig. 1C).

For breakfast and all meals combined, there were no statistically significant treatment differences between the postmeal URLi and mealtime lispro group in PPG iAUC for any time interval. The results in the postmeal URLi versus mealtime URLi group were comparable for all breakfast meal comparisons, but for all meals combined, the iAUC_0–2h_ and iAUC_0–3h_ were statistically significantly higher in the postmeal URLi versus the mealtime URLi group (Fig. 1C).

### Postmeal glucose excursions

Consistent with the AGPs, mealtime URLi statistically significantly reduced postmeal glucose excursions compared to mealtime lispro up to 3 h for all meals combined (Table 1). There were no statistically significant differences in postmeal glucose excursions between postmeal URLi and mealtime lispro but postmeal URLi demonstrated statistically significantly increased postmeal glucose excursions compared to mealtime URLi up to 2 h for all meals combined. There were no statistically significant treatment differences in time to postmeal maximum glucose excursion (Table 1).

**Table:** 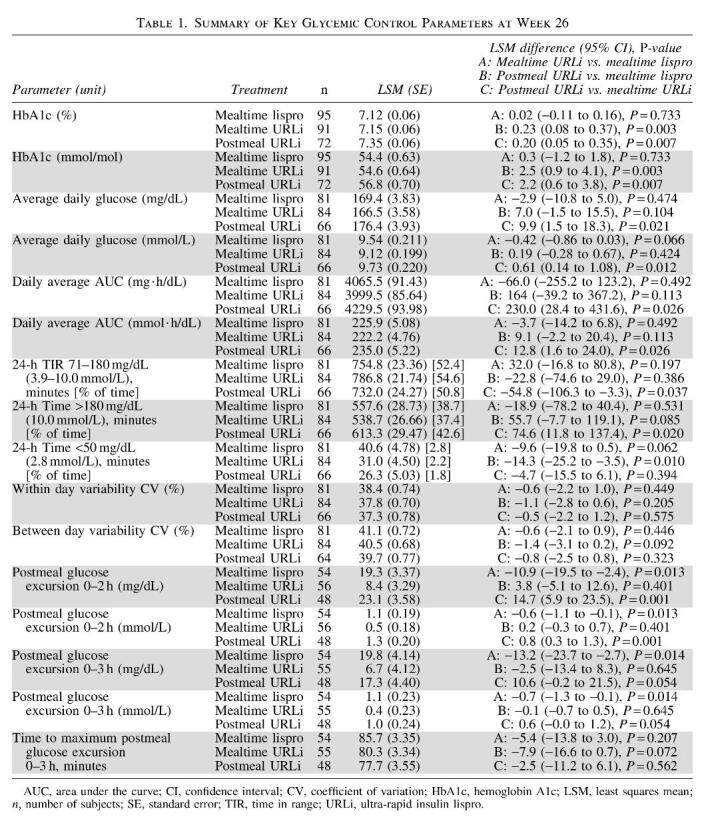

### Time in ranges

**FIG. 2.**
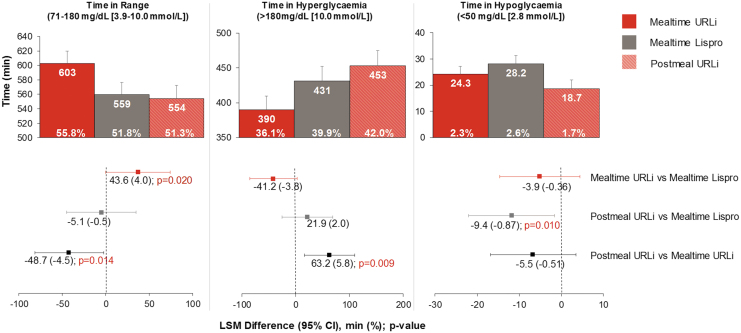
Daytime time in ranges at week 26 (6:00 am–midnight). CI, confidence interval; LSM, least squares mean.

During the daytime period, mealtime URLi use was associated with increased TIR (71–180 mg/dL) compared to mealtime lispro (LSM difference = 43.6 min; *P* = 0.020) (Fig. 2). Lowering of daytime time above range (>180 mg/dL) and time below range with mealtime URLi did not achieve statistical significance compared to mealtime lispro (LSM differences of −41.2 min and −3.9 min, respectively) (Fig. 2). Postmeal URLi was associated with a significantly decreased time in hypoglycemia (<50 mg/dL) compared to mealtime lispro (LSM difference = −9.4 min; *P* = 0.010) (Fig. 2); however, it was also associated with a significantly increased time above range compared to mealtime URLi (LSM difference = 63.2 min; *P* = 0.009) (Fig. 2).

During the 24-h period, mealtime URLi statistically significantly increased TIR (71–180 mg/dL) and statistically significantly decreased time above range compared to postmeal URLi (Table 1). Postmeal URLi statistically significantly decreased time in hypoglycemia (≤70, <54, and <50 mg/dL) compared to mealtime lispro (Table 1; Supplementary Table S4).

**FIG. 3.**
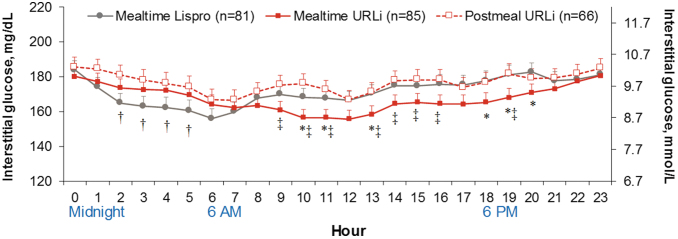
Hourly average glucose during continuous glucose monitoring at week 26. Data are LSM ± SE. *P < 0.05 for mealtime URLi versus mealtime lispro, ^†^*P* < 0.05 for postmeal URLi versus mealtime lispro, ^‡^*P* < 0.05 for postmeal URLi versus mealtime URLi. *n*, number of subjects; SE, standard error.

During the nighttime period, there were no statistically significant treatment differences between all three treatment arms in TIR (71–180 mg/dL) (Supplementary Table S4). Postmeal URLi statistically significantly increased time above range compared to mealtime lispro (Supplementary Table S4). Mealtime URLi and postmeal URLi statistically significantly decreased time in hypoglycemia (≤70 and <54 mg/dL) compared to mealtime lispro (Supplementary Table S4); however, these improvements in hypoglycemia risk were associated with higher hourly average glucose during the nighttime period (Fig. 3).

### Mean daily glucose profiles

Figure 3 illustrates hourly average glucose by CGM for each treatment group over 24 h at week 26. At multiple timepoints throughout the daytime period, mealtime URLi resulted in statistically significantly lower daytime glucose compared to mealtime lispro consistent with the demonstrated improvements in PPG, but overnight glucose was consistently, but not statistically significantly, higher than mealtime lispro. Postmeal URLi resulted in statistically significantly higher glucose at multiple timepoints during the nocturnal period compared to mealtime lispro.

Average daily glucose was similar in the mealtime URLi and mealtime lispro groups, but statistically significantly higher in the postmeal URLi group compared to the mealtime URLi group (Table 1). Mealtime URLi resulted in similar daily average AUC glucose compared to mealtime lispro for the daytime, nighttime, and 24-h period (Table 1; Supplementary Fig. S1). Postmeal URLi resulted in statistically significantly higher daily AUC compared to mealtime lispro for the nighttime period and compared to mealtime URLi for the daytime and 24-h period (Table 1; Supplementary Fig. S1).

### Glucose variability

Conventional measures of within-day, between-day, and overall glycemic variability (such as coefficient of variation, SD, mean absolute glucose excursion [MAGE], mean of daily differences) defined for the 24-h, daytime, and nighttime periods showed that mealtime URLi had similar variability to mealtime lispro (Table 1; Supplementary Table S5). Postmeal URLi had greater variability by some of these measures, compared to mealtime lispro (low blood glucose index) and mealtime URLi (MAGE, high blood glucose index) (Table 1; Supplementary Table S5).

## Discussion

URLi and lispro demonstrated similar HbA1c reduction in the main PRONTO-T1D study^7^ and the same was true in this CGM substudy. However, HbA1c provides an incomplete picture of daily fluctuations in glycemic control, such as PPG and TIR, which are important to patients.^12^ In a large study of patients with type 1 diabetes and type 2 diabetes (*n* = 1200), Heller et al. found that increased severity and frequency of acute postprandial hyperglycemia is associated with greater burden and experience of symptoms and can negatively impact daily functioning of patients.^13^ CGM-based measures such as TIR provide additional information that patients can use to understand how their insulin, meals, and exercise affect glycemic control. TIR was also identified as one of the “biggest drivers of improved diabetes management and mindset,” and ranked only behind food choice as having the biggest impact on daily life in a survey of 3461 people with type 1 diabetes or type 2 diabetes.^14^

In this substudy of PRONTO-T1D, blinded CGM was offered to a subgroup of patients in each of the three treatment groups to provide additional information related to PPG control, TIR, and glycemic variability. The primary objective of this CGM substudy was achieved as mealtime URLi use was characterized by lower iAUC_0–2h_ when compared to mealtime lispro, and this superiority was also shown for the comparisons of the iAUC_0–3h_ and iAUC_0–4h_. Results from the ambulatory PPG assessments demonstrating also improved PPG control and reduced PPG excursions during treatment with mealtime URLi are consistent with observations from both the mixed-meal tolerance test and 10-point SMBG profiles in the main study.^7^

One potential limitation of these comparisons of mealtime URLi and lispro relates to the inability to optimize timing of administration relative to the start of the meal based on their unique time-action profiles and still maintain the rigor afforded by double-blind treatment. Although administration of insulin lispro is recommended within 15 min before a meal, it is generally recognized that optimal effects are seen when rapid insulin analogs are given 15–20 min before a meal, survey data from the T1D Exchange examining the time of prandial insulin administration relative to meals (“On average, when do you take your insulin in relation to mealtime?” asked on March 8, 2014) showed that only 16.3% of patients who responded indicated that they inject 15 min before meals (T1D Exchange, email communication, April 29, 2020).

Results from a prior phase 1 test meal study in patients with T1D demonstrated that URLi performed better than lispro (glucose AUC_0–2h_) when both were administered 15 min before the meal.^15^ Based on these considerations, both mealtime URLi and lispro were administered 0–2 min before the start of the meal in the current study to facilitate double blinding and to minimize potential postprandial hypoglycemia risk with URLi in an outpatient setting.

The improvements in PPG control observed in mealtime URLi patients were accomplished without an increase in time below range versus the mealtime lispro group. In fact, mealtime URLi was associated with an increased time spent in target glycemic range during the daytime period, which is the period when prandial insulins are typically used. This observation is especially notable because the overall glycemic control achieved in these treatment groups was good and quite similar.

Mealtime URLi patients also spent statistically significantly less time in hypoglycemia during the nighttime period (<50 mg/dL [2.8 mmol/L] and (≤70 mg/dL [3.9 mmol/L]) compared to the mealtime lispro group; although these findings were associated with numerically higher nocturnal hourly average glucose during treatment with mealtime URLi. This contrasts with the main study, where no statistically significant treatment differences were detected in the rate or incidence of nocturnal hypoglycemia^7^ and were very likely related to the greater sensitivity of CGM for the assessment of hypoglycemia occurrence,^16^ which does not depend on patient's symptoms for detection. Visual inspection of AGPs representing the 4-h postmeal intervals and the mean glucose profiles (Fig. 1A, B) shows reduced glucose excursions postmeals (confirmed by postmeal AUC measurements).

Postmeal URLi did not demonstrate statistically significant differences in PPG control or TIR compared to mealtime lispro; however, it resulted in larger postmeal excursions, less time in target glycemic range, and more time in hyperglycemia compared to mealtime URLi, especially during the daytime. CGM monitoring provided the opportunity to detect increases in time spent in hyperglycemia or decreases in time spent in hypoglycemia in the postmeal URLi arm that were not possible with conventional blood glucose monitoring and may help guide the appropriate use of postmeal dosing. While postmeal URLi administration was shown to be a safe and efficacious option in the PRONTO-T1D study, it may not be optimal in most patients and should probably be reserved for special situations (e.g., when premeal glucose is low or meal consumption is unpredictable).

In general, this CGM substudy provided insight into ambient glucose in an unbiased, ad libitum feeding, ambulatory setting that adds to the body of evidence that URLi administered at the start of the meal provides superior PPG control compared to mealtime lispro. These AGPs also provide insight into the time patients spent in target range and a thorough, reassuring assessment of hypoglycemia that was not dependent on patient self-reporting.

The results of this substudy also suggest possible avenues for further optimization of dosing with URLi. For example, Figure 3 clearly demonstrates improved daytime glycemia with mealtime URLi compared to lispro. However, mean glucose levels were lower with lispro during the overnight period. It is therefore possible that additional titration of basal insulin or use of a closed-loop insulin delivery system could improve overall glycemic control with the use of URLi. These possibilities should be explored in future studies.

In conclusion, during the daytime period, URLi administered immediately before meals resulted in better PPG control, increased time spent in target range, and no increases in time spent in hypoglycemia compared to mealtime lispro even though both groups reported similar HbA1c after 26 weeks of treatment. URLi administered 20 min after the start of the meal was similar to mealtime lispro, but less optimal than mealtime URLi in managing PPG when these insulins were administered immediately before meals. Results from this CGM substudy support key observations from the main study and suggest that URLi administered before the start of meals may favorably influence PPG and other glycemic parameters “beyond HbA1c” that are important to patients.

## Supplementary Material

Supplemental data

Supplemental data

Supplemental data

Supplemental data

Supplemental data

Supplemental data
