# Supplementary material for: Ultra-Rapid Lispro Improves Postprandial Glucose Control and Time in Range in Type 1 Diabetes Compared to Lispro: PRONTO-T1D Continuous Glucose Monitoring Substudy
Source: Diabetes Technol Ther. 2020 Nov 9;22(11):853–60. doi: 10.1089/dia.2020.0129 (PMC7698997; doi:10.1089/dia.2020.0129)
Supplement: Supplemental data [file Supp_TableS4.pdf]

Supplementary Table S4. Time in Ranges at Week 26

Parameter	*Treatment*	*n*	*Time in minutes, LSM (SE)*	*LSM difference (95% CI),* P*-value A: Mealtime URLi vs. mealtime lispro B: Postmeal URLi vs. mealtime lispro C: Postmeal URLi vs. mealtime URLi*	*% of time, LSM*
Nighttime					
TIR 70–180 mg/dL	Mealtime lispro	81	189.2 (8.26)	A: −1.3 (−18.3 to 15.8), *P* = 0.885	52.5
[3.9–10.0 mmol/L]	Mealtime URLi	85	187.9 (7.62)	B: −7.2 (−25.4 to 11.0), *P* = 0.437	52.2
	Postmeal URLi	66	182.0 (8.52)	C: −5.9 (−23.9 to 12.1), *P* = 0.516	50.5
TIR 71–180 mg/dL	Mealtime lispro	81	187.6 (8.21)	A: −0.9 (−17.9 to 16.1), *P* = 0.918	52.1
[3.9–10.0 mmol/L]	Mealtime URLi	85	186.7 (7.58)	B: −6.6 (−24.7 to 11.5), *P* = 0.475	51.9
	Postmeal URLi	66	181.0 (8.47)	C: −5.7 (−23.6 to 12.2), *P* = 0.532	50.3
Time >180 mg/dL	Mealtime lispro	81	133.0 (9.67)	A: 12.5 (−7.1 to 32.2), *P* = 0.211	36.9
[10.0 mmol/L]	Mealtime URLi	85	145.5 (8.89)	B: 22.1 (1.0 to 43.3), *P* = 0.040	40.4
	Postmeal URLi	66	155.1 (9.81)	C: 9.6 (−11.2 to 30.4), *P* = 0.363	43.1
Time ≤70 mg/dL	Mealtime lispro	81	37.6 (4.07)	A: −11.5 (−20.0 to −2.9), *P* = 0.009	10.5
[3.9 mmol/L]	Mealtime URLi	85	26.2 (3.84)	B: −14.1 (−23.2 to −4.9), *P* = 0.003	7.3
	Postmeal URLi	66	23.5 (4.24)	C: −2.6 (−11.7 to 6.5), *P* = 0.573	6.5
Time <54 mg/dL	Mealtime lispro	81	15.7 (2.63)	A: −5.8 (−11.4 to −0.3), *P* = 0.039	4.4
[3.0 mmol/L]	Mealtime URLi	85	9.9 (2.48)	B: −6.6 (−12.6 to −0.7), *P* = 0.030	2.8
	Postmeal URLi	66	9.1 (2.76)	C: −0.8 (−6.7 to 5.1), *P* = 0.797	2.5
Time <50 mg/dL	Mealtime lispro	81	12.6 (2.30)	A: −5.6 (−10.5 to −0.8), *P* = 0.023	3.5
[2.8 mmol/L]	Mealtime URLi	85	7.0 (2.17)	B: −5.4 (−10.6 to −0.2), *P* = 0.043	1.9
	Postmeal URLi	66	7.2 (2.41)	C: 0.2 (−4.9 to 5.4), *P* = 0.925	2.0
Daytime					
TIR 70–180 mg/dL	Mealtime lispro	81	563.2 (17.62)	A: 44.0 (7.0 to 81.1), *P* = 0.020	52.1
[3.9–10.0 mmol/L]	Mealtime URLi	84	607.2 (16.54)	B: −5.1 (−44.6 to 34.3), *P* = 0.798	56.2
	Postmeal URLi	66	558.0 (18.45)	C: −49.2 (−88.4 to −9.9), *P* = 0.014	51.7
Time ≤70 mg/dL	Mealtime lispro	81	88.7 (7.59)	A: −2.4 (−18.6 to 13.7), *P* = 0.766	8.2
[3.9 mmol/L]	Mealtime URLi	84	86.3 (7.16)	B: −15.7 (−32.9 to 1.5), *P* = 0.074	8.0
	Postmeal URLi	66	73.0 (8.00)	C: −13.2 (−30.4 to 3.9), *P* = 0.130	6.8
Time <54 mg/dL	Mealtime lispro	81	36.0 (3.89)	A: −3.3 (−11.5 to 5.0), *P* = 0.436	3.3
[3.0 mmol/L]	Mealtime URLi	84	32.7 (3.67)	B: −10.3 (−19.1 to −1.5), *P* = 0.022	3.0
	Postmeal URLi	66	25.7 (4.10)	C: −7.0 (−15.8 to 1.8), *P* = 0.117	2.4
24-h period					
TIR 70–180 mg/dL	Mealtime lispro	81	760.3 (23.59)	A: 32.0 (−17.2 to 81.3), *P* = 0.201	52.8
[3.9–10.0 mmol/L]	Mealtime URLi	84	792.3 (21.95)	B: −23.6 (−75.9 to 28.8), *P* = 0.376	55.0
	Postmeal URLi	66	736.7 (24.50)	C: −55.6 (−107.6 to −3.6), *P* = 0.036	51.2
Time ≤70 mg/dL	Mealtime lispro	81	127.1 (10.15)	A: −14.1 (−35.5 to 7.3), *P* = 0.195	8.8
[3.9 mmol/L]	Mealtime URLi	84	113.0 (9.57)	B: −30.0 (−53.0 to −7.1), *P* = 0.011	7.8
	Postmeal URLi	66	97.0 (10.62)	C: −15.9 (−38.8 to 7.0), *P* = 0.172	6.7
Time <54 mg/dL	Mealtime lispro	81	51.7 (5.73)	A: −9.1 (−21.3 to 3.0), *P* = 0.140	3.6
[3.0 mmol/L]	Mealtime URLi	84	42.5 (5.41)	B: −16.6 (−29.6 to −3.6), *P* = 0.013	3.0
	Postmeal URLi	66	35.1 (6.03)	C: −7.5 (−20.4 to 5.5), *P* = 0.258	2.4

CI, confidence interval; TIR, time in range.
