# Supplementary material for: Ultra-Rapid Lispro Improves Postprandial Glucose Control and Time in Range in Type 1 Diabetes Compared to Lispro: PRONTO-T1D Continuous Glucose Monitoring Substudy
Source: Diabetes Technol Ther. 2020 Nov 9;22(11):853–60. doi: 10.1089/dia.2020.0129 (PMC7698997; doi:10.1089/dia.2020.0129)
Supplement: Supplemental data [file Supp_FigS1-TableS1.pdf]

Supplementary Data

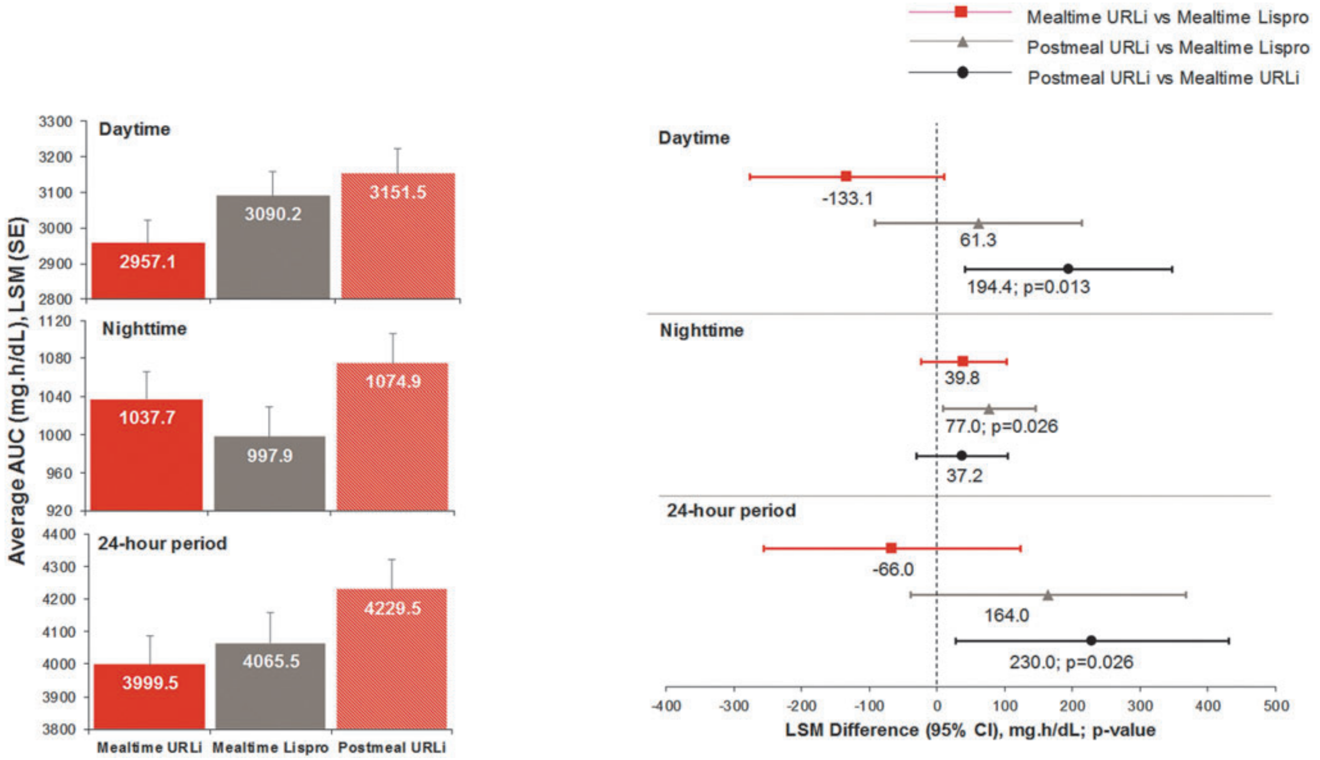

SUPPLEMENTARY FIG. S1. Daily average area under the glucose concentration curve at week 26. AUC, area under the curve; CI, confidence interval; LSM, least squares mean; SE, standard error; URLi, ultra-rapid insulin lispro.

SUPPLEMENTARY TABLE S1. PATIENT DISPOSITION FOR THE CONTINUOUS GLUCOSE MONITORING COHORT

Patients	*Mealtime lispro*	*Mealtime URLi*	*Postmeal URLi*	*Total*
Randomized, *n*	116	111	86	313
Received at least 1 dose of study treatment and wore the CGM device during at least 1 collection period (either baseline or postbaseline), *n*	99	97	73	269
Discontinued the study early, *n* (%)	4 (3.4)	3 (2.7)	1 (1.2)	8 (2.6)
Discontinued treatment early during the treatment period, *n* (%)	6 (5.2)	6 (5.4)	4 (4.7)	16 (5.1)

CGM, continuous glucose monitoring; *n*, number of subjects; URLi, ultra-rapid insulin lispro.
