# Supplementary material for: Ultra-Rapid Lispro Improves Postprandial Glucose Control and Time in Range in Type 1 Diabetes Compared to Lispro: PRONTO-T1D Continuous Glucose Monitoring Substudy
Source: Diabetes Technol Ther. 2020 Nov 9;22(11):853–60. doi: 10.1089/dia.2020.0129 (PMC7698997; doi:10.1089/dia.2020.0129)
Supplement: Supplemental data [file Supp_TableS2.pdf]

Supplementary Table S2. Baseline and Demographic Characteristics for the Continuous Glucose Monitoring Cohort

Parameter	Mealtime lispro (N=99)	Mealtime URLi (N=97)	Postmeal URLi (N=73)	Total (N=269)	P*
Sex, *n* (%)					
Female	41 (41.4)	44 (45.4)	27 (37.0)	112 (41.6)	0.547
Male	58 (58.6)	53 (54.6)	46 (63.0)	157 (58.4)	
Age (years)					
Mean (SD)	46.18 (12.68)	44.57 (14.17)	42.27 (13.52)	44.54 (13.50)	0.172
Minimum, maximum	20, 73	24, 77	20, 71	20, 77	
Age group, *n* (%)					
<65 years	92 (92.9)	88 (90.7)	68 (93.2)	248 (92.2)	0.795
≥65 years	7 (7.1)	9 (9.3)	5 (6.8)	21 (7.8)	
Race, *n* (%)					
Asian	1 (1.0)	1 (1.0)	1 (1.4)	3 (1.1)	0.369
Black or African American	0 (0.0)	0 (0.0)	2 (2.7)	2 (0.7)	
White	95 (96.0)	91 (93.8)	66 (90.4)	252 (93.7)	
Ethnicity, *n* (%)					
Hispanic or Latino	10 (10.1)	10 (10.3)	9 (12.3)	29 (10.8)	0.527
Not Hispanic or Latino	89 (89.9)	86 (88.7)	62 (84.9)	237 (88.1)	
Country, *n* (%)					
Australia	4 (4.0)	5 (5.2)	5 (6.8)	14 (5.2)	0.938
Germany	14 (14.1)	8 (8.2)	8 (11.0)	30 (11.2)	
Italy	2 (2.0)	3 (3.1)	2 (2.7)	7 (2.6)	
Mexico	2 (2.0)	2 (2.1)	4 (5.5)	8 (3.0)	
New Zealand	4 (4.0)	3 (3.1)	1 (1.4)	8 (3.0)	
Poland	20 (20.2)	24 (24.7)	11 (15.1)	55 (20.4)	
Puerto Rico	3 (3.0)	3 (3.1)	1 (1.4)	7 (2.6)	
Spain	7 (7.1)	7 (7.2)	6 (8.2)	20 (7.4)	
United States	43 (43.4)	42 (43.3)	35 (47.9)	120 (44.6)	
BMI (kg/m^2)					
Mean (SD)	27.73 (4.02)	27.21 (3.76)	28.24 (4.36)	27.68 (4.03)	0.250
Minimum, maximum	18.90, 35.50	19.00, 35.00	18.60, 37.00	18.60, 37.00	
Duration of diabetes (years)					
Mean (SD)	22.00 (12.33)	21.40 (12.37)	18.16 (10.09)	20.74 (11.85)	0.087
Minimum, maximum	1.56, 52.03	2.00, 63.27	1.71, 45.32	1.56, 63.27	
HbA1c, mean (SD)					
Study screening (%)†	8.00 (0.66)	7.98 (0.64)	8.00 (0.67)	7.99 (0.65)	0.967
Study screening (mmol/mol)†	63.95 (7.24)	63.72 (7.02)	63.97 (7.29)	63.87 (7.15)	
Baseline (%)	7.28 (0.68)	7.23 (0.61)	7.24 (0.66)	7.25 (0.65)	0.893
Baseline (mmol/mol)	56.02 (7.45)	55.56 (6.69)	55.68 (7.21)	55.76 (7.09)	
FSG, mean (SD)					
Study entry (mg/dL)‡	174.32 (69.98)	170.47 (78.99)	175.53 (73.30)	173.26 (74.00)	0.894
Study entry (mmol/L)‡	9.68 (3.88)	9.46 (4.38)	9.74 (4.07)	9.62 (4.11)	
Baseline (mg/dL)§	129.38 (43.37)	123.46 (46.17)	131.85 (43.06)	127.94 (44.28)	0.446
Baseline (mmol/L)§	7.18 (2.41)	6.86 (2.56)	7.32 (2.39)	7.10 (2.46)	
Prestudy basal insulin, *n* (%)					
Insulin glargine	66 (66.7)	67 (69.1)	52 (71.2)	185 (68.8)	0.704
NPH	2 (2.0)	3 (3.1)	1 (1.4)	6 (2.2)	
Insulin degludec	13 (13.1)	16 (16.5)	14 (19.2)	43 (16.0)	
Insulin detemir	18 (18.2)	11 (11.3)	6 (8.2)	35 (13.0)	
Lead-in basal insulin, *n* (%)					
Insulin glargine QD	60 (60.6)	48 (49.5)	39 (53.4)	147 (54.6)	0.292
Insulin glargine BID	6 (6.1)	14 (14.4)	10 (13.7)	30 (11.2)	
Insulin degludec QD	33 (33.3)	35 (36.1)	24 (32.9)	92 (34.2)	
Personal CGM/FGM use, *n* (%)					
Yes	27 (27.3)	21 (21.6)	24 (32.9)	72 (26.8)	0.259

**P*-value for overall treatment effect.
†Lispro, *N*=98; mealtime URLi, *N*=94, postmeal URLi, *N*=72; total, *N*=264.
‡Lispro, *N*=98; total, *N*=268.
§Lispro, *N*=97; mealtime URLi, *N*=94, postmeal URLi, *N*=72; total, *N*=263.
BID, twice daily; BMI, body mass index; FGM, flash glucose monitoring; FSG, fasting serum glucose; HbA1c, hemoglobin A1c; *N*, number of subjects in population; QD, once daily; SD, standard deviation.
