# Supplementary material for: Ultra-Rapid Lispro Improves Postprandial Glucose Control and Time in Range in Type 1 Diabetes Compared to Lispro: PRONTO-T1D Continuous Glucose Monitoring Substudy
Source: Diabetes Technol Ther. 2020 Nov 9;22(11):853–60. doi: 10.1089/dia.2020.0129 (PMC7698997; doi:10.1089/dia.2020.0129)
Supplement: Supplemental data [file Supp_TableS3.pdf]

Supplementary Table S3. Summary of Key Glycemic Control Parameters in the Continuous Glucose Monitoring Cohort Versus the Full Study Population

	CGM cohort			*Full study population*		
	Mealtime lispro (N = 99)	*Mealtime URLi (N = 97)*	*Postmeal URLi (N = 73)*	*Mealtime lispro (N = 442)*	*Mealtime URLi (N = 451)*	*Postmeal URLi (N = 329)*
Hypoglycemia incidence (≤70 mg/dL [3.9 mmol/L]), *n* (%) from randomization to week 26						
Documented symptomatic	92 (92.93)	92 (94.85)	66 (90.41)	404 (91.40)	414 (91.80)	293 (89.06)
All documented	99 (100.00)	97 (100.00)	72 (98.63)	440 (99.55)	449 (99.56)	325 (98.78)
Non-nocturnal	93 (93.94)	94 (96.91)	70 (95.89)	421 (95.25)	433 (96.01)	311 (94.53)
Nocturnal	79 (79.80)	78 (80.41)	57 (78.08)	328 (74.21)	344 (76.27)	251 (76.29)
Hypoglycemia rate (events/patient/30 days), (≤70 mg/dL [3.9 mmol/L]), LSM (SE) at week 26						
Documented symptomatic	4.93 (0.53)	5.51 (0.59)	5.62 (0.61)	4.94 (0.26)	4.70 (0.24)	4.93 (0.27)
All documented	8.33 (0.56)	8.83 (0.68)	9.01 (0.71)	8.14 (0.29)	7.54 (0.27)	8.16 (0.31)
Non-nocturnal	5.98 (0.49)	6.45 (0.62)	6.77 (0.62)	5.75 (0.25)	5.20 (0.23)	5.92 (0.28)*
Nocturnal	1.17 (0.17)	1.17 (0.15)	1.01 (0.15)	1.16 (0.09)	1.11 (0.08)	1.03 (0.08)
Insulin dose (U/kg), LSM (SE) at week 26						
Basal‡	0.39 (0.01)	0.38 (0.01)	0.38 (0.01)	0.37 (0.00)	0.38 (0.00)	0.38 (0.00)
Bolus	0.45 (0.02)	0.44 (0.02)	0.44 (0.02)	0.41 (0.01)	0.43 (0.01)	0.42 (0.01)
Total	0.84 (0.02)	0.84 (0.02)	0.82 (0.02)	0.79 (0.01)	0.81 (0.01)	0.80 (0.01)
HbA1c (%), LSM (SE)						
Baseline	7.28 (0.07)	7.24 (0.07)	7.24 (0.08)	7.33 (0.03)	7.34 (0.03)	7.36 (0.04)
Week 26	7.12 (0.06)	7.15 (0.06)	7.35 (0.06)*,†	7.29 (0.03)	7.21 (0.03)	7.42 (0.04)*,†
Change at week 26	–0.13 (0.06)	–0.11 (0.06)	0.10 (0.06)	–0.05 (0.03)	–0.13 (0.03)	0.08 (0.04)
HbA1c (mmol/mol), LSM (SE)						
Baseline	56.0 (0.72)	55.6 (0.73)	55.7 (0.83)	56.7 (0.34)	56.7 (0.34)	56.9 (0.40)
Week 26	54.4 (0.63)	54.6 (0.64)	56.8 (0.70)*,†	56.1 (0.34)	55.3 (0.33)	57.6 (0.39)*,†
Change at week 26	–1.4 (0.63)	–1.2 (0.64)	1.0 (0.70)	–0.6 (0.34)	–1.4 (0.33)	0.8 (0.39)

*$P < 0.05$ postmeal URLi versus mealtime URLi.

†$P < 0.05$ postmeal URLi versus mealtime lispro.

‡Patients were stratified according to basal insulin use during the study. In the main study cohort, formal treatment-by-subgroup interactions analyses were performed for type of basal insulin. No significant treatment by subgroup interactions were identified in analyses of HbA1c or hypoglycemia rate.

LSM, least squares mean; SE, standard error.
