# Supplementary material for: Ultra-Rapid Lispro Improves Postprandial Glucose Control and Time in Range in Type 1 Diabetes Compared to Lispro: PRONTO-T1D Continuous Glucose Monitoring Substudy
Source: Diabetes Technol Ther. 2020 Nov 9;22(11):853–60. doi: 10.1089/dia.2020.0129 (PMC7698997; doi:10.1089/dia.2020.0129)
Supplement: Supplemental data [file Supp_TableS5.pdf]

Supplementary Table S5. Glycemic Variability Parameters at Week 26

Parameter (unit)	*Treatment*	*n*	*LSM (SE)*	*LSM difference (95% CI),* P*-value* *A: Mealtime URLi vs. mealtime lispro* *B: Postmeal URLi vs. mealtime lispro* *C: Postmeal URLi vs. mealtime URLi*
Within day variability for 24 h				
SD mg/dL	Mealtime lispro	81	63.6 (1.50)	A: −1.2 (−4.4 to 2.0), *P* = 0.449
	Mealtime URLi	84	62.3 (1.41)	B: 0.6 (−2.8 to 4.0), *P* = 0.725
	Postmeal URLi	66	64.2 (1.58)	C: 1.8 (−1.5 to 5.2), *P* = 0.286
SD mmol/L	Mealtime lispro	81	3.53 (0.083)	A: −0.07 (−0.24 to 0.11), *P* = 0.449
	Mealtime URLi	84	3.46 (0.078)	B: 0.03 (−0.15 to 0.22), *P* = 0.725
	Postmeal URLi	66	3.56 (0.088)	C: 0.10 (−0.09 to 0.29), *P* = 0.286
IQR mg/dL	Mealtime lispro	81	95.3 (3.12)	A: 0.3 (−6.3 to 6.9), *P* = 0.935
	Mealtime URLi	84	95.6 (2.93)	B: −0.8 (−7.8 to 6.2), *P* = 0.818
	Postmeal URLi	66	94.5 (3.29)	C: −1.1 (−8.1 to 5.9), *P* = 0.758
IQR mmol/L	Mealtime lispro	81	5.29 (0.173)	A: 0.02 (−0.35 to 0.38), *P* = 0.935
	Mealtime URLi	84	5.31 (0.163)	B: −0.05 (−0.44 to 0.34), *P* = 0.818
	Postmeal URLi	66	5.25 (0.183)	C: −0.06 (−0.45 to 0.33), *P* = 0.758
MAGE mg/dL	Mealtime lispro	81	163.2 (3.96)	A: −3.4 (−11.8 to 5.0), *P* = 0.429
	Mealtime URLi	84	159.8 (3.74)	B: 7.0 (−2.0 to 15.9), *P* = 0.127
	Postmeal URLi	66	170.2 (4.17)	C: 10.3 (1.4 to 19.3), *P* = 0.024
MAGE mmol/L	Mealtime lispro	81	9.07 (0.220)	A: −0.19 (−0.66 to 0.28), *P* = 0.429
	Mealtime URLi	84	8.88 (0.208)	B: 0.39 (−0.11 to 0.88), *P* = 0.127
	Postmeal URLi	66	9.45 (0.232)	C: 0.57 (0.08 to 1.07), *P* = 0.024
LBGI	Mealtime lispro	81	2.09 (0.174)	A: −0.24 (−0.61 to 0.12), *P* = 0.190
	Mealtime URLi	84	1.85 (0.164)	B: −0.50 (−0.90 to −0.11), *P* = 0.012
	Postmeal URLi	66	1.59 (0.182)	C: −0.26 (−0.65 to 0.13), *P* = 0.192
HBGI	Mealtime lispro	81	10.02 (0.628)	A: −0.57 (−1.88 to 0.73), *P* = 0.389
	Mealtime URLi	84	9.44 (0.589)	B: 0.87 (−0.52 to 2.27), *P* = 0.219
	Postmeal URLi	66	10.89 (0.649)	C: 1.45 (0.06 to 2.83), *P* = 0.041
Between day variability for 24 h				
SD mg/dL	Mealtime lispro	81	67.4 (1.53)	A: −1.3 (−4.6 to 1.9), *P* = 0.423
	Mealtime URLi	84	66.1 (1.45)	B: 0.9 (−2.6 to 4.4), *P* = 0.628
	Postmeal URLi	64	68.3 (1.63)	C: 2.2 (−1.3 to 5.7), *P* = 0.218
SD mmol/L	Mealtime lispro	81	3.75 (0.085)	A: −0.07 (−0.25 to 0.11), *P* = 0.423
	Mealtime URLi	84	3.67 (0.080)	B: 0.05 (−0.15 to 0.24), *P* = 0.628
	Postmeal URLi	64	3.79 (0.091)	C: 0.12 (−0.07 to 0.32), *P* = 0.218
MODD mg/dL	Mealtime lispro	81	77.5 (1.98)	A: −2.9 (−7.1 to 1.3), *P* = 0.174
	Mealtime URLi	84	74.6 (1.87)	B: 0.5 (−4.0 to 5.0), *P* = 0.823)
	Postmeal URLi	64	78.0 (2.12)	C: 3.4 (−1.1 to 7.9), *P* = 0.136
MODD mmol/L	Mealtime lispro	81	4.31 (0.110)	A: −0.16 (−0.40 to 0.07), *P* = 0.174
	Mealtime URLi	84	4.15 (0.104)	B: 0.03 (−0.22 to 0.28), *P* = 0.823
	Postmeal URLi	64	4.34 (0.118)	C: 0.19 (−0.06 to 0.44), *P* = 0.136
Overall variability for 24 h				
SD mg/dL	Mealtime lispro	81	72.0 (1.65)	A: −0.6 (−4.1 to 2.9), *P* = 0.749
	Mealtime URLi	84	71.5 (1.56)	B: 0.2 (−3.5 to 3.9), *P* = 0.910
	Postmeal URLi	66	72.2 (1.74)	C: 0.8 (−2.9 to 4.5), *P* = 0.677
SD mmol/L	Mealtime lispro	81	4.00 (0.092)	A: −0.03 (−0.23 to 0.16), *P* = 0.749
	Mealtime URLi	84	3.97 (0.087)	B: 0.01 (−0.20 to 0.22), *P* = 0.910
	Postmeal URLi	66	4.01 (0.097)	C: 0.04 (−0.16 to 0.25), *P* = 0.677
IQR mg/day	Mealtime lispro	81	101.2 (3.33)	A: 0.3 (−6.7 to 7.4), *P* = 0.929
	Mealtime URLi	84	101.5 (3.13)	B: −1.2 (−8.7 to 6.3), *P* = 0.755
	Postmeal URLi	66	100.0 (3.50)	C: −1.5 (−9.0 to 6.0), *P* = 0.691
IQR mmol/L	Mealtime lispro	81	5.62 (0.185)	A: 0.02 (−0.37 to 0.41), *P* = 0.929
	Mealtime URLi	84	5.64 (0.174)	B: −0.07 (−0.48 to 0.35), *P* = 0.755
	Postmeal URLi	66	5.56 (0.195)	C: −0.08 (−0.50 to 0.33), *P* = 0.691
LBGI	Mealtime lispro	81	2.09 (0.174)	A: −0.25 (−0.62 to 0.12), *P* = 0.184
	Mealtime URLi	84	1.84 (0.164)	B: −0.51 (−0.91 to −0.12), *P* = 0.011
	Postmeal URLi	66	1.58 (0.182)	C: −0.26 (−0.66 to 0.13), *P* = 0.186
HBGI	Mealtime lispro	81	10.00 (0.628)	A: −0.56 (−1.87 to 0.74), *P* = 0.397
	Mealtime URLi	84	9.44 (0.589)	B: 0.88 (−0.52 to 2.27), *P* = 0.218
	Postmeal URLi	66	10.88 (0.649)	C: 1.44 (0.05 to 2.83), *P* = 0.042

HBGI, high blood glucose index; IQR, interquartile range; LBGI, low blood glucose index; MAGE, mean absolute glucose excursion; MODD, mean of daily differences.
