# Supplementary material for: Ultra-Rapid Lispro Improves Postprandial Glucose Control and Time in Range in Type 1 Diabetes Compared to Lispro: PRONTO-T1D Continuous Glucose Monitoring Substudy
Source: Diabetes Technol Ther. 2020 Nov 9;22(11):853–60. doi: 10.1089/dia.2020.0129 (PMC7698997; doi:10.1089/dia.2020.0129)
Supplement: Supplemental data [file Supp_TableS6.pdf]

Supplementary Table S6. List of Investigators by Country

Australia	
	Claire Morbey, Adam Roberts, Richard Simpson, Stephen Stranks
Germany	
	Hans-Peter Kempe, Jörg Lüdemann, Ludger Rose, Thomas Schaum, Heike Schlichthaar, Alexander Segner
Italy	
	Maria Gisella Cavallo, Francesco Dotta, Marina Scavini
Mexico	
	Jose Gerardo Gonzalez Gonzalez
New Zealand	
	Richard Carroll, Simon Young
Poland	
	Katarzyna Cypryk, Edward Franek, Elwira Gromniak, Katarzyna Jusiak, Monika Lukaszewicz, Maciej Malecki, Beata Matyjaszek-Matuszek, Henryk Rudzki, Ewa Skokowska, Dariusz Sowinski
Spain	
	Alberto Aliaga Verdugo, Cristobal Morales, Francisco Tinahones Madueño
United States	
	Altagracia Alcantara-Gonzalez, Anuj Bhargava, Timothy Bailey, Bruce Bode, Anna Chang, Ronald Chochinov, Angel Comulada, David Fitz-Patrick, Satish Garg, Martha Gomez Cuellar, Barry Horowitz, Thomas Jones, Leslie Klaff, Sam Lerman, Kathryn Lucas, James Magee, Wendell Miers, Frank Mikell, Paul Norwood, Ramon Ortiz-Carrasquillo, Kerem Ozer, Betsy Palal, John Parker, Antonio Pinero Pilona, John Reed, Julio Rosenstock, Robert Silver, Larry Stonesifer, Carl Vance, Mark Warren, Daniel Weiss, and Michelle Welch
